# Deep Characterization of the Human Antibody Response to Natural Infection Using Longitudinal Immune Repertoire Sequencing*[Fn FN2]

**DOI:** 10.1074/mcp.RA119.001633

**Published:** 2019-11-25

**Authors:** Erin M. Mitsunaga, Michael P. Snyder

**Affiliations:** Department of Genetics, Stanford University School of Medicine, Stanford, California 94305

**Keywords:** Antibodies, cell sorting, RNA SEQ, blood, gene expression, molecular biology, personalized medicine, B cell subsets, class switching, complementarity-determining region 3, human immune repertoire, longitudinal profiling

## Abstract

Human antibody response studies are largely restricted to periods of high immune activity (*e.g.* vaccination). To comprehensively understand the healthy B cell immune repertoire and how this changes over time and through natural infection, we conducted immune repertoire RNA sequencing on flow cytometry-sorted B cell subsets to profile a single individual's antibodies over 11 months through two periods of natural viral infection. We found that 1) a baseline of healthy variable (V) gene usage in antibodies exists and is stable over time, but antibodies in memory cells consistently have a different usage profile relative to earlier B cell stages; 2) a single complementarity-determining region 3 (CDR3) is potentially generated from more than one VJ gene combination; and 3) IgG and IgA antibody transcripts are found at low levels in early human B cell development, suggesting that class switching may occur earlier than previously realized. These findings provide insight into immune repertoire stability, response to natural infections, and human B cell development.

## Abstract (precis)

We conducted targeted immune repertoire RNA sequencing on flow cytometry-sorted human B cell subsets to longitudinally profile how the healthy human antibody response changes through time and natural infections. Our results indicate that antibodies have stable variable (V) gene usage profiles based on antigen experience. We additionally observed that one VJ gene combination can give rise to more than one complementarity-determining region 3 (CDR3) sequence. Unexpectedly, we also found that isotype switching may occur earlier than previously thought.

## Graphical Abstract

**Figure:**
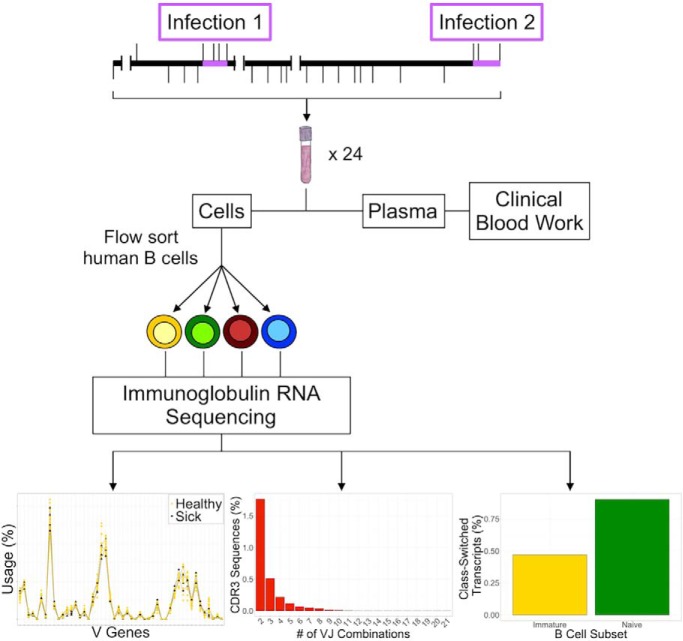

- Longitudinal monitoring of B cell subsets shows baseline antibody gene expression.
- A single, given CDR3 sequence can arise from more than one VJ gene combination.
- A healthy individual's V gene usage is stable irrespective of infection and subset.
- Surprisingly, class-switched antibodies can occur early in human B cell development.

Understanding human health requires a multi-faceted approach that has traditionally involved measuring cells, small molecules, and proteins in blood and recording this information in conjunction with physiological measurements and self-reported symptoms. Recent advances in sequencing technologies and computational analyses now enable us to specifically probe the human immune repertoire transcriptome, which provides a new window into immune function. This surge in data collection has led to an increasing focus on personalized medicine, where an individual's personal and medical histories are combined to create a comprehensive outlook on health status and inform both preventive medical care and medical treatment (1). What has remained unclear is the stability of a healthy human immune repertoire over time and how natural infections affect this normal immune baseline.

Prior studies centered on analyzing the human B cell repertoire have often focused on either a specific immunological challenge (2, 3, 4) or the B cell subset-specificity of complementarity-determining region 3s (CDR3s), the hypervariable region of the antibody protein responsible for determining antigen-binding specificity (5); these regions are formed by random combinations of the variable (V), diversity (D), and joining (J) gene segments (6, 7, 8). However, having a focused approach has specific limitations. In the case of disease-associated analyses, most experiments were performed on bulk B cells, resulting in the loss of valuable information about cellular subsets. Whereas experiments designed to analyze B cell subset-specific CDR3^1^ properties avoid this issue, the sampling resolution was usually restricted to a single blood draw from participating individuals, resulting in a static perspective on an otherwise dynamic system. Studies that combine both multi-time point sampling of an immune challenge event on sorted B cell subsets are becoming more common (9, 10, 11, 12), but understanding the B cell repertoire of healthy individuals over time (13) and through infection is quite rare. As a result, our understanding of the antibody repertoire across different B cell subsets, its stability over time, how it changes during natural viral infection is limited.

To address this, we longitudinally profiled an individual's immune repertoire in a subset-specific manner through two natural infection events. This approach has several advantages: 1) having access to a motivated individual allows higher sample number and consistency; 2) large sample numbers allow for increased confidence in identifying patterns in fluctuating signals while giving higher resolution to potentially low-level or rare observations; 3) the longer an individual is studied, the greater the chance of observing both healthy and natural infection periods, enabling the study of altered conditions in the same person (1); and 4) having well-defined periods of infection (*e.g.* elevated hs-CRP, white blood cell, and neutrophil percentage levels) enables correlation of particular immune repertoire changes to either healthy or aberrant function.

Additionally, we sorted bulk peripheral blood B cells into four distinct subsets because: 1) the majority of total B cells are from the naïve subset (14), leading to an overrepresentation of this population in data collected from unsorted samples; 2) bulk B cell characterization masks subset-specific data that differentiates between B cell developmental stages and antigen naïvete *versus* experience (*i.e.* immature and naïve *versus* memory and plasmacyte cells (7)); and 3) examining antibody sequence data of B cells at different developmental stages through both time and differing health statuses shows how the immune repertoire is affected and what changes are made during responses, especially relative to original antigenic sin (15).

Here, we analyzed targeted RNA sequencing data derived from the CDR3s of flow cytometry-sorted healthy human B cell subsets through two natural viral infections over the course of 11 months. Through our approach, we characterized B cell response dynamics at high resolution in both healthy and disease states of the same individual. We found that 1) antibody repertoires have both common and unique features across the different B cell subsets over time in a healthy individual; 2) a given CDR3 could be generated from more than one VJ gene segment combination; 3) an individual's V gene usage preferences are stable through time and natural infection across B cell subsets; and 4) class-switched antibodies (*i.e.* IgG and IgA) are found at low levels in early human B cell development, suggesting that class switching occur may earlier than previously thought. These findings provide fundamental insights into human immune system development, stability and response.

## EXPERIMENTAL PROCEDURES

### 

#### 

##### Experimental Design and Statistical Rationale

The study subject was a healthy 59-year-old Caucasian male with a well-documented medical history; the Stanford Administrative Panel of Human Subjects in Medical Research approved his participation. At each of 24 time points over a period of 11 months, 10 ml of blood were collected via venipuncture into EDTA Vacutainer tubes (BD Biosciences, San Jose, CA) after fasting between the hours of 7:00 am and 10:00 am Pacific Standard Time (PST) to standardize the effects of circadian rhythm on leukocytes (16). In addition, the subject communicated any feelings of illness experienced, including but not limited to fever, sore throat, body aches, and general malaise. During these time points, nasopharyngeal swabs were collected from the subject and genotyped using the GenMark Diagnostics eSensor Respiratory Viral Panel kit and cartridges (Carlsbad, CA), which are FDA cleared for 14 common respiratory virus types and subtypes. Clinical blood work done by the Stanford Clinical and Translational Research Unit (CTRU) were also utilized to further confirm the subject's self-reported health status.

All raw sequence data was submitted to iRepertoire, Inc. (Huntsville, AL) as FASTQ files for analysis with their customized, in-house pipeline. Raw data was analyzed using the previously described iRmap program (17, 18). Briefly, sequence reads were de-multiplexed according to barcode sequences at the 5′ end of reads from the B-cell receptor (BCR) heavy constant regions. Reads were then trimmed according to their base qualities with a 2-base sliding window. If either quality value in this window was lower than 20, these sequence stretches from the window to 3′ end were trimmed from the original read. Trimmed paired-end reads were joined through overlapping alignment with a modified Needleman-Wunsch algorithm. If paired forward and reverse reads in the overlapping region were not perfectly matched, both forward and reverse reads were thrown out without further consideration. The merged reads were mapped using a Smith-Waterman algorithm to germline V, D, J, and C reference sequences downloaded from the IMGT web site (19). To define the CDR3 region, the position of CDR3 boundaries of reference sequences from the IMGT database were migrated onto reads through mapping results, and the resulting CDR3 regions were extracted and bioinformatically translated into amino acids.

For the purposes of this study, we further filtered the iRepertoire-analyzed data by eliminating 1) any predicted non-functional CDR3 sequences and 2) any CDR3s that were not observed four or more times from further downstream analysis per the company's recommendation. Where only three of the four subsets are used, we purposefully chose to leave out the plasmacyte subset because of low cell numbers. Additionally, time points H1, H2?, H3, and H9 were also excluded from some of the analyses because of low sequencing depth. To increase the probability of sequencing all cells according to a Poisson distribution, all samples were minimally sequenced to a depth of five reads per cell based on the company's recommendation. Visualization of the resulting nucleic and amino acid sequences was done in IgBLAST through the National Center for Biotechnology Information (NCBI) using default parameters (20). All statistical analyses from the resulting data were done in R (version 3.3.2 (21)) and all graphs generated from these analyses were done with the R package *ggplot2* (version 2.2.1; 22).

To determine if there were significant differences in CDR3 amino acid length, the Kolmogorov-Smirnov (K-S) test was used. In the case of amino acid usage, the χ^2^ test was utilized. To compare between healthy and sick V gene distributions, the Kruskal-Wallis test was used. For subset differences between V gene usage, the χ^2^ test was implemented. For both the comparison of isotype distributions between immature and naïve B cells, the Wilcoxon signed rank test was used whereas the K-S test was applied to analyze isotype distribution between the immature, naïve, and memory B cell subsets. Where necessary, *p* values were adjusted with the Benjamini-Hochberg multiple hypothesis correction. Corrected *p* values less than 0.05 were considered significant.

##### Sample Preparation

After collection, each blood sample underwent centrifugation at 400 × *g* for 10 min at 4 °C. All plasma was removed and replaced with an equivalent volume of cold 2.5 mm solution of EDTA in PBS (EDTA-PBS) (Thermo Fisher Scientific, Waltham, MA). The resulting reconstituted blood was then prepared for fluorescence-activated cell sorting (FACS).

The staining panel was designed to positively select for four B cell subsets based on developmental stages known to be present in human peripheral blood (14). Both compensation beads (BD Biosciences; Thermo Fisher Scientific - Invitrogen) and aliquots of reconstituted blood were used as negative, single color, and fluorescence-minus-one (FMO) controls and underwent the same staining procedure as described below.

All reconstituted blood cell samples and the appropriate controls were initially stained for 15 min at room temperature with the amine-reactive Zombie Aqua L/D dye (BioLegend, San Diego, CA) to discriminate between living and dead cells. The following subset-specific cell surface markers (identified by clone and fluorochrome) from BD Biosciences were then added to the samples to enable sorting of live B cell populations: anti-CD10 (HI10a; PE-CF594), anti-CD19 (SJ25C1; APC-Cy7), anti-CD27 (M-T271; PE-Cy7), anti-CD38 (HIT2; BV421), and anti-CD45 (HI30; APC) with the addition of Brilliant Buffer per the manufacturer's instructions. The stained samples were incubated on ice in the dark for 30 min. An excess volume of cold 2.5 mm EDTA-PBS with 10% FBS (Thermo Fisher Scientific) was used to wash the stained cells. All tubes were then centrifuged at 490 × *g* for 10 min at 4 °C. To remove red blood cells, BD Pharmlyse Lysing Buffer (BD Biosciences) was added and the samples were again incubated on ice in the dark for 30 min. All tubes were then centrifuged at 490 × *g* for 10 min at 4 °C. All samples were then washed in an excess of cold 2.5 mm EDTA-PBS with 10% FBS and centrifuged at 490 × *g* for 5 min at 4 °C. Pellets were resuspended in an appropriate volume of cold 2.5 mm EDTA-PBS with 10% FBS and stored in the dark at 4 °C for sorting within 24 h of sample collection.

##### Flow Cytometry

All samples were sorted on a BD FACSAria II flow cytometer at the Stanford Shared FACS Facility. Compensation and gating strategy were performed using unstained, single color, and FMO cells in addition to unstained and single-color beads. Bulk B cells were phenotypically defined as CD45^+^ CD19^+^; the four B cell subsets were further defined as follows: immature (CD10^+^ CD27^−^ CD38^−^), naïve (CD10^−^ CD27^−^ CD38^−^), memory (CD10^−^ CD27^+^ CD38^−^) and plasmacyte (CD10^−^ CD27^+^ CD38^+^). All live subset sorting events were recorded (supplemental Table S1). After sorting, all samples were centrifuged at 490 × *g* for 5 min at 4 °C and the supernatant was discarded. Cell pellets were resuspended in Buffer RLT Plus with β-mercaptoethanol from the AllPrep RNA/DNA Micro Kit (Qiagen, Valencia, CA) and centrifuged through QIAshredder spin columns. All cell lysates were stored at −80 °C for library preparation.

##### Targeted Immune Repertoire Sequencing

RNA was isolated from all four subsets at each time point according to the manual instructions for the AllPrep RNA/DNA Micro Kit (Qiagen). The purified RNA from all four subsets was quality checked with a Nanodrop 2000 (Thermo Fisher Scientific) before use as template material in the creation of cDNA libraries for sequencing.

To analyze the CDR3 sequences, RT-PCR reactions were conducted using a proprietary set of commercially available nested sequence-specific primers from iRepertoire Inc. covering human VH genes (forward primers) and constant C-primers (reverse primers). The Illumina paired-end sequencing communal primer B is linked to each forward inside V primer whereas the Illumina paired-end sequencing communal primer A and a barcode sequence of 6 nucleotides are linked to the reverse inside C primer. In brief, cDNA was reverse transcribed from the total RNA samples from each B cell subset using a mixture of forward V and reverse C primers and reagents from the OneStep RT-PCR kit (Qiagen).

The first round of RT-PCR was performed at: 50 °C, 41 min; 95 °C, 15 min; 94 °C, 30 s, 63 °C, 5 min, 72 °C, 30 s, for 10 cycles; 94 °C, 30 s, 72 °C, 3 min, for 10 cycles; 72 °C, 5 min. After the first round of PCR, 2 μl of PCR1 product was added to 48 μl of PCR2 Multiplex Mix (Qiagen). The second round of PCR (PCR2) was performed with a set of communal primers that complete the Illumina adaptor sequences as: 95 °C, 15 min; 94 °C, 30 s, 72 °C, 90 s, for 30 cycles; 72 °C, 5 min. After PCR2, 50 μl of PCR2 product was run on a 2% agarose gel to assess amplification success. Amplicon bands were excised and gel purified using a Qiaquick Gel Purification kit (Qiagen) per manufacturer's instructions.

The completed cDNA libraries were assessed for quality and quantified on the Qubit Fluorometer (Thermo Fisher Scientific) and the Agilent 2100 Bioanalyzer (Agilent, Santa Clara, CA) before getting pooled and submitted to the Stanford Center for Genomics and Personalized Medicine for paired-end sequencing at 2 × 250 bp on the Illumina MiSeq platform.

##### Examination of Published Data

To further investigate our observation of more than one VJ gene combination creating a single CDR3 sequence, we downloaded published publicly available data sets from four different studies (2, 23–25). We chose to utilize each study's processed data to enable more robust comparisons to our data set and minimize issues concerning differences in experimental design and analysis pipeline. The data sets were then filtered for only CDR3 sequences with identified V and J genes. The remaining sequences were then consolidated to determine how many VJ gene combinations resulted in the same CDR3s.

## RESULTS

### 

#### 

##### Elevated Levels of High Sensitivity CRP, White Blood Cells, and Neutrophil Percentage Define Periods of Infection

**Fig. 1.**
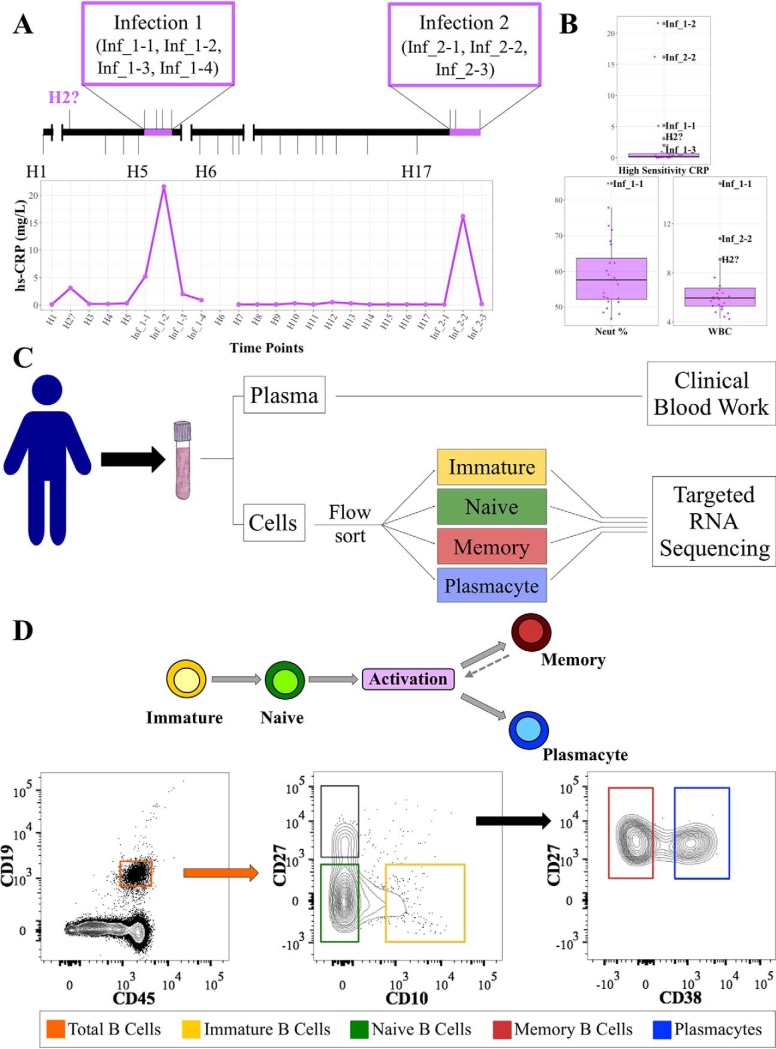
**Defining health status by combining blood test results with self-reporting.** *A*, Timeline showing the sampling schedule with self-reported illness periods in relation to elevated hs-CRP measurements. *B*, Normalized values of the three parameters measured in the clinical blood work that showed significant elevation during infected time points. See also supplemental Fig. S1. *C*, Blood was drawn from our subject 24 times over the course of an 11-month period. The samples were separated into plasma and cellular fractions and processed according to the flow chart. *D*, The top panel shows a generalized schematic of human B cell development. The bottom panel illustrates the flow cytometry gating scheme used to sort B cell subsets.

To examine the immune repertoire in different B-cell subsets over time and during natural infection, we collected 24 whole blood samples from a single adult male over an 11-month period (Fig. 1*A*, upper panel). At each time point, a complete blood count with differential test was performed in addition to recording the subject's health-related events and a panel of clinical measurements. During this time, the subject experienced two distinct periods of infection as determined by integrating self-reporting and elevated high sensitivity C-reactive protein (hs-CRP) levels based on clinical blood work (26; Fig. 1*A*, lower panel). Elevated white blood cell levels and neutrophil percentage also positively correlated with the subject's self-reported health status and hs-CRP levels (*p* ≤ 0.05), further confirming the periods of illness (27; Fig. 1*B* and supplemental Fig. S1).

To identify the virus associated with each infection, nasopharyngeal swabs were collected from the subject. In each case, the subject was positively confirmed as infected by human rhinovirus (see Experimental Procedures) and both incidents were classified as natural viral infections.

For each time point, we purified four developmental stages of B cells (immature, naïve, memory, and plasmacyte) by fluorescence-activated cell sorting (FACS) using subset-specific cell surface markers based on a modification of Perez-Andres *et al.* (14; Figs. 1*C* and 1*D*). Targeted immune repertoire sequencing on these sorted B cell populations was performed to a minimum depth of five reads per cell for further downstream analysis. We then used a series of different analyses to examine the immune repertoire across chain types, B cell subsets, and time. We focused on 1) CDR3 features at the predicted protein level; 2) overall diversity of CDR3 predicted peptide sequences; 3) the number of VJ gene segment combinations that contribute to each CDR3; 4) V gene usage during natural infections; and 5) isotype usage through health and illness.

##### Differences in CDR3 Characteristics Are Chain-Specific

**Fig. 2.**
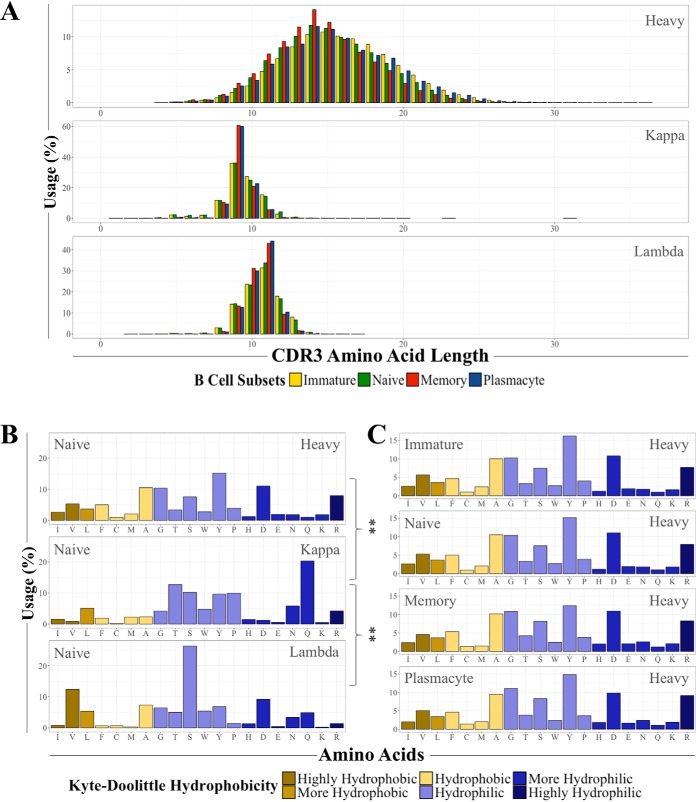
**Differences in CDR3 characteristics are chain-specific.** *A*, Distributions of all four B cell subset CDR3 amino acid lengths separated by chain type across all 24 time points. See also supplemental Table S2. *B*, A representative comparison of amino acid usage of the different chain types using the naïve B cell subset. Asterisks indicate a χ^2^ test *p* value ≤ 0.01 after multiple hypothesis correction. See also supplemental Tables S3 and S4. *C*, The heavy chain amino acid usage of the four different B cell subsets over all 24 time points are shown.

To first examine CDR3 characteristics through time and immune perturbation, we compared the CDR3 amino acid length of the heavy, kappa, and lambda chains across all time points and subsets. As expected, we found a significant difference in amino acid length between the heavy chain and both light chain types (28; Fig. 2*A* and supplemental Table S2); however, for each chain type and B cell subset the length did not significantly vary across time or health status (not shown). These results are consistent with the findings of Mroczek *et al.* (7).

To determine whether the chain-based differences extended to amino acid composition, we compared the predicted amino acid usage of the three chain types between all four subsets. For the naïve, memory, and plasmacyte cell types, there was both a significant and consistent difference between the amino acids of the kappa chain and the heavy and lambda chains, particularly in glutamine usage (Fig. 2*B*; supplemental Tables S3 and S4); the difference for the heavy and lambda chains was not significant, nor were any of the immature cell chain comparisons. Additionally, we examined if there was a difference in predicted amino acid usage for each of the chains across the B cell subsets and found there was no significant difference (Fig. 2*C*). We also did not detect any significant differences across time or during either of the viral infections. Taken together, these results suggest the observed differences in the basic molecular features of CDR3s are specific to chain type and do not show significant changes over time or through natural viral infections.

##### CDR3s Show High Diversity Across B Cell Subsets

After observing these chain-specific differences, we next examined the appearance of any given predicted CDR3 amino acid sequence and the maintenance of those observed CDR3s through B cell maturation as a function of 1) time; 2) natural infections; and 3) antigen experience (*i.e.* immature and naïve *versus* memory and plasmacyte populations).

**Fig. 3.**
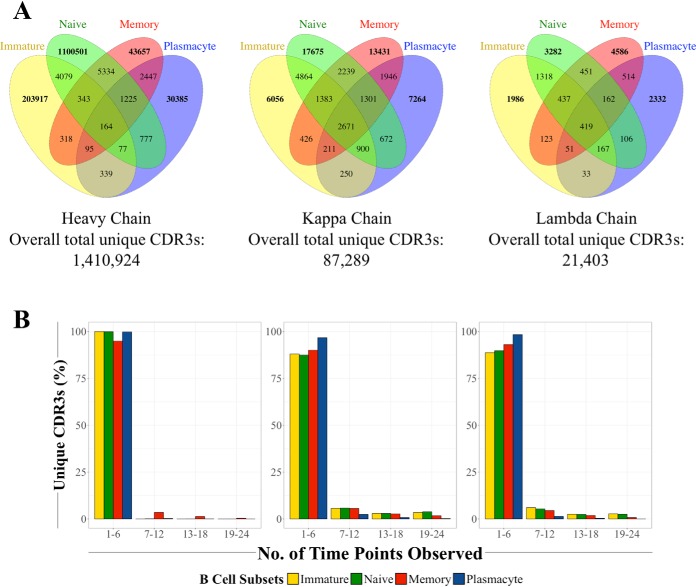
**There are high levels of unique CDR3 diversity among all three chains and between all four B cell subsets.** *A*, Venn diagrams showing the number of overlapping CDR3s between B cell subsets in the heavy, kappa, and lambda chains, respectively. See also supplemental Table S5. *B*, Most unique CDRs are observed in less than 25% of all of the time points, with the majority appearing once.

To better understand the basal diversity of CDR3 sequences through time, we first filtered the data to obtain unique CDR3 sequences for each time point and B cell subset (*i.e.* each observed CDR3 sequence was counted only once per time point irrespective of actual abundance). We then examined the presence of each unique CDR3 sequence in each cell type and chain (supplemental Table S5). We found that the vast majority of these sequences were subset-specific, with the proportion of unique CDR3s that were common to all four B cell subsets as 0.01%, 3.1%, and 2.0% for the heavy, kappa, and lambda chains, respectively (Fig. 3*A*). We also observed that the number of heavy chain unique CDR3s in each subset closely follows the proportion of each cell type in the total B cell population (supplemental Table S5). Analysis of the unique CDR3s by health status (*i.e.* healthy *versus* ill) did not reveal any significant differences in the distribution of unique CDR3s (not shown).

We also examined the commonality of unique CDR3 sequences through each developmental stage. Interestingly, CDR3s from the four subsets and all three chain types predominantly appeared in the range of one to six time points (Fig. 3*B*); within that range, the bulk of those CDR3s only appeared once (not shown). All these observations indicate that 1) monitoring unique CDR3s through time can potentially serve as a measurement proxy for the relative proportions of B cell subsets and 2) there is a high amount of diversity in peripheral blood B cell heavy and light chain CDR3s throughout B cell development.

##### A Single Heavy Chain CDR3 Can Be Made from More Than One VJ Gene Combination

Because the four distinct B cell subsets had consistently high heterogeneity of CDR3 sequences, we examined if the underlying VJ gene combinations in each cell type also reflected this variability. To this end, we determined the median numbers of both unique CDR3s and VJ combinations observed over all 24 time points for each cell type; the resulting numbers indicated there are multiple potential CDR3s for each VJ combination with wide variation among the different chains and cell types (supplemental Table S6; the median CDR3 and VJ combinations range from 524 to 48,092 and 49 to 260, respectively).

**Fig. 4.**
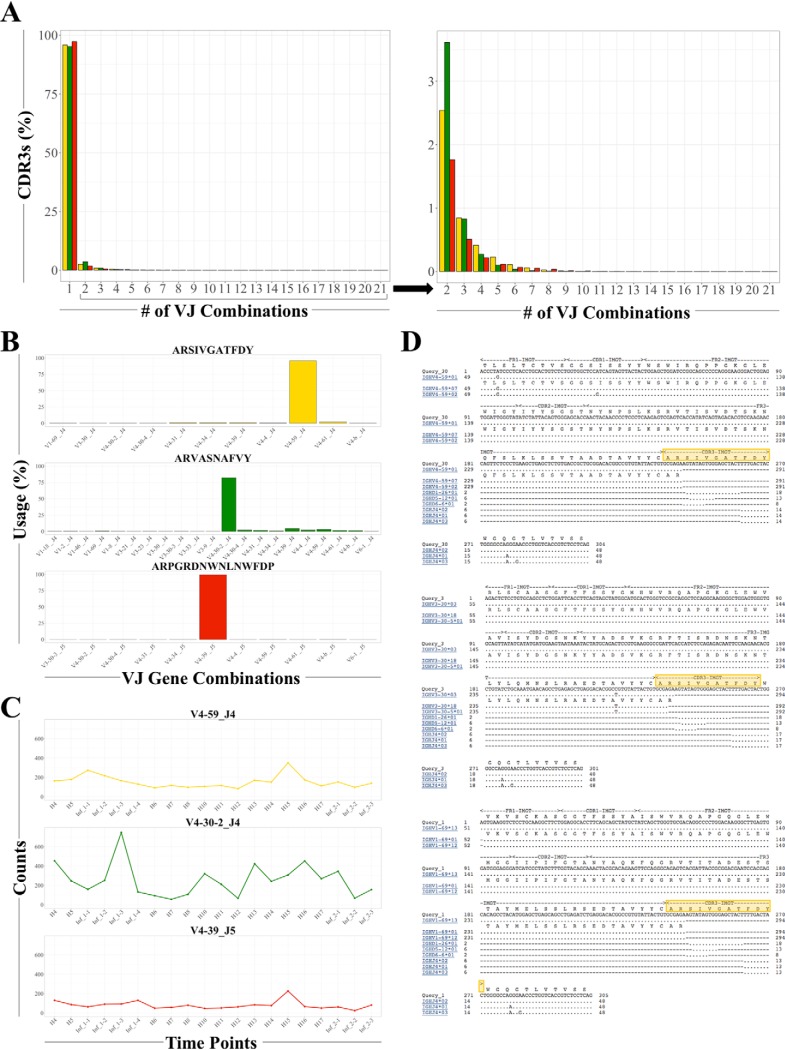
**Multiple CDR3s can be made from a single VJ combination while a single CDR3 can be made from several different VJ combinations.** *A*, The majority of unique CDR3s are made by a single VJ combination. The right panel is a closer view of the distribution of unique CDR3s made from more than one VJ combination. The immature subset is shown in yellow, the naive subset in green, and the memory subset in red. See also supplemental Fig. S2 and supplemental Table S6. *B*, The representative heavy chain CDR3 amino acid sequences for each subset are displayed in yellow (immature), green (naïve) and red (memory) with the percent observed of each VJ gene combination on the *y* axis and the VJ gene combination on the *x* axis. See also supplemental Figs. S2 and S4. *C*, The heavy chain VJ gene combination most frequently used in (B) was tracked over time to quantify the number of CDR3s that used the same combination. The CDR3 counts observed are on the *y* axis and the time points are on the *x* axis. The line graphs are shown in yellow (immature subset), green (naïve subset), and red (memory subset). See also supplemental Fig. S2. *D*, IgBLAST results for three different VJ combinations that resulted in the same immature B cell CDR3 amino acid sequence ARSIVGATFDY (highlighted in yellow). See also supplemental Fig. S3.

We then surveyed all unique CDR3s and all possible VJ combinations to identify how frequently multiple VJ combinations contribute to multiple CDR3s. We examined the heavy chains further because they have the highest possible combinatorial diversity based on the number of V and J genes (see Discussion). For the immature, naïve, and memory B cell subsets, whereas the vast majority of CDR3s are made from one VJ combination (the highest diversity is in the naïve subset; Fig. 4*A*, left panel), there are some CDR3s that are made from many VJ combinations (Fig. 4*A*, right panel).

Based on unique predicted amino acid sequences, we then selected the most highly expressed CDR3 from each subset and chain type to quantify the number of VJ combinations used (Fig. 4*B* and supplemental Fig. S2*B*; based on cDNA sequences). Although one VJ combination dominated in each CDR3 sequence, we found that multiple VJ combinations were used. We then chose the most frequently used VJ combination and plotted how many different CDR3s also used that VJ combination across each time point (Fig. 4*C* and supplemental Fig. S2*C*). In each subset and chain type, that combination was used in each time point and hundreds of different CDR3s, indicating that multiple combinations of VJ genes can make the same CDR3 amino acid sequence.

To further investigate the plausibility of multiple VJ combinations yielding the same predicted amino acid CDR3 sequence, we compared both the nucleic and amino acid sequences via IgBLAST. The resulting alignments were consistent with both the initial assignments and the possibility that distinct V genes can combine with J genes and result in the same CDR3 sequence at both the nucleic and amino acid levels across not only the naïve subset, but the immature and memory populations as well (Fig. 4*D* and supplemental Figs. S3*A* and S3*B*). The confluence of both nucleic acid and protein sequence here is quite surprising, particularly given the junction sequences randomly added upon rearrangement.

We also investigated whether one VJ combination can generate multiple CDR3 sequences for each of the three B cell developmental stages. As expected, the same VJ combination can be used for distinct CDR3s (supplemental Fig. S2*A*).

Overall, we observed that a single unique CDR3 can be generated from several different VJ gene combinations and a single VJ combination can give rise to a wide variety of CDR3s.

##### An Individual's Antigen Exposure Is Associated with Preferential V Gene Usage

Although the multiplicity of VJ gene usage in CDR3s across the B cell subsets was clearly observed, we also noted that, in each heavy chain subset exemplar, V and J genes from the same family were preferentially used with the exception of J5 in the memory subset (supplemental Fig. S4). As a result, we sought to determine whether there were any preferences in V and/or J gene usage.

**Fig. 5.**
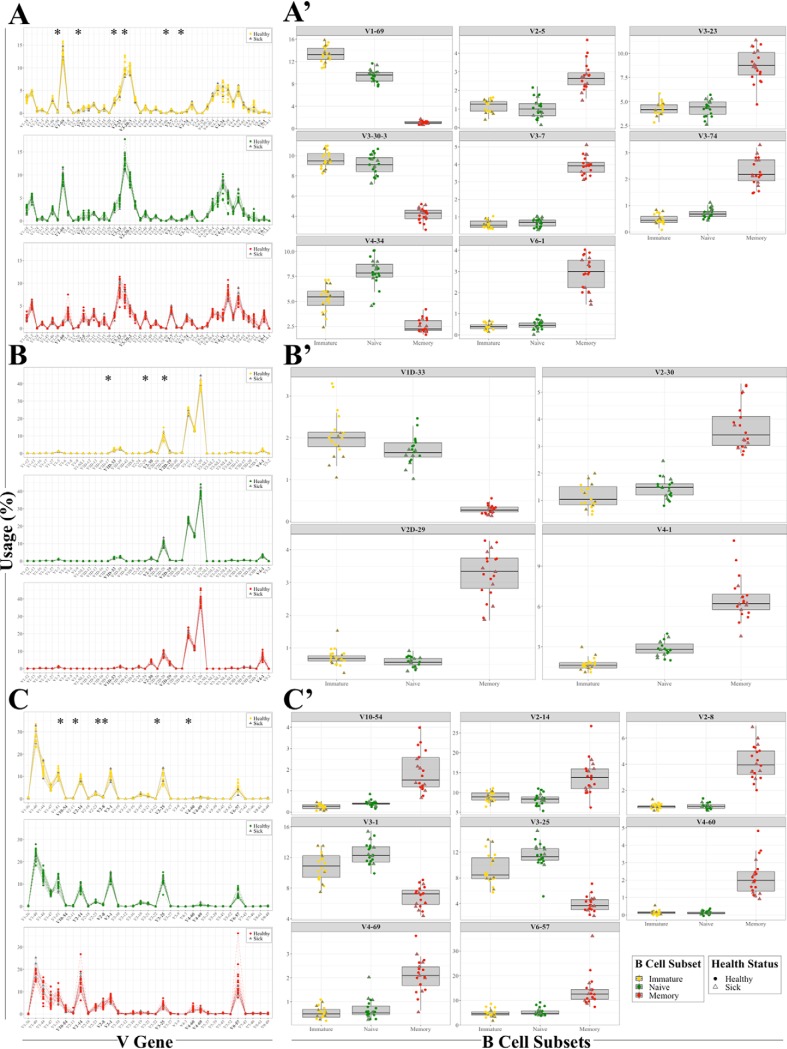
**V gene usage preferences are shaped by an individual's antigen exposure.** *A*, *B*, and *C*, V gene usage of the heavy, kappa, and lambda chains at the immature, naïve, and memory subset level for 20 time points. The asterisks indicate significant differences in V gene usage between B cell subsets with a corrected *p* ≤ 0.05 as determined by χ^2^ test. *A*′, *B*′, and *C*′, V genes that were determined to have significantly different usage are shown for all three subsets and the three chain types. Healthy time points are shown as closed circles and infected time points are plotted as open triangles. (*A–C*′) See also supplemental Fig. S4 and supplemental Table S7.

We first examined how health state and antigen experience contribute toward V gene usage. Expressed V genes from the immature, naïve, and memory B cell subsets across all three chain types were separated into healthy and infected time points based on our definition of infection (self-reporting with hs-CRP levels > 1.0 mg/L) and the resulting distributions were graphed for comparison (Fig. 5*A*, 5*B*, and 5*C*). All the time points showed remarkable similarity for V gene usage, regardless of health status. While the overall usage distributions did not reveal any statistically significant differences based on health, several V genes did pass our threshold for significance after multiple hypothesis correction, as indicated by the asterisks (Fig. 5*A*, 5*B*, and 5*C*). Further inspection of those V genes revealed that the immature and naïve subsets have similar biases in usage, which is inversely related to their usage in the memory subset (Fig. 5*A*′, 5*B*′, and 5*C*′). These results suggest that V gene usage preferences are set in early in development (*i.e.* immature cells) and that V gene usage frequency shifts in memory cells. Importantly, V gene use does not change upon natural infection in any of the B cell subsets relative to healthy time points (Fig. 5*A*′, 5*B*′, and 5*C*′).

We next examined J gene usage across the B cell subsets through time. We found a clear bias toward the J4 gene across all subsets at each time point with significant differences in J6 gene usage between all subsets (supplemental Fig. S4 and supplemental Table S7). These features were evident across both healthy and infection time points. However, it is important to note that this observation is most relevant to measuring overall CDR3 J gene usage as opposed to the J gene preference of any given CDR3. An example of a different preferential J usage for a CDR3 is the high prevalence of J5 in the representative memory CDR3 amino acid sequence ARPGRDNWNLNWFDP as opposed to the J4 which is dominant overall in the cell types (Fig. 4*B* and supplemental Fig. S4).

In conclusion, these results indicate that V gene usage is highly stable across time for each B cell subset, regardless of health status.

##### Consistent Immune Repertoire Monitoring Reveals Evidence of Class Switching in Both Immature and Naïve Human B Cells

**Fig. 6.**
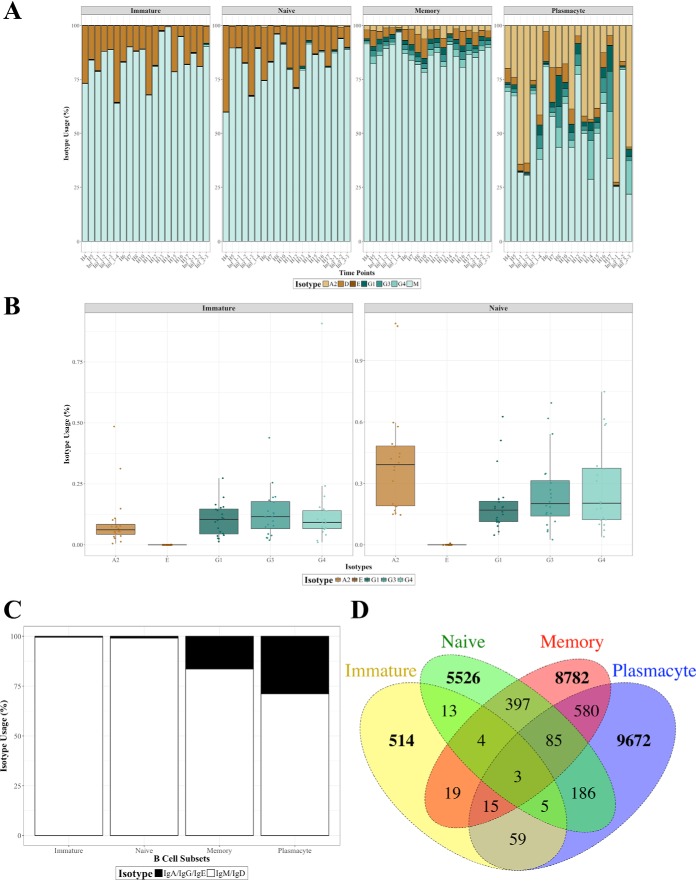
**Isotype switching observed in human immature and naïve B cells.** *A*, The isotype distributions of each subset through the natural infections. *B*, Usage of IgA2, IgG1, IgG3, and IgG4 in the immature and naïve subsets are significantly different from zero as determined by the Wilcoxon signed rank. *C*, Levels of IgA, IgG, and IgE in the antigen-inexperienced immature and naïve subsets relative to the antigen-experienced memory and plasmacyte subsets. *D*, Venn diagrams showing the counts of overlapping IgA, IgG, or IgE CDR3s between all four B cell subsets. (*A–D*) See also supplemental Fig. S5.

Only IgM and IgD are expected in immature and naïve cells, whereas memory cells and plasmacytes are expected to contain additional isotypes: IgA, IgG, and possibly IgE. To determine if this was reflected in our study, we analyzed isotype expression within each population as a function of health through time (Fig. 6*A*).

Unexpectedly, there were isotypes other than IgM and IgD present in both the immature and naïve B cell subsets. A close examination of the other 5 isotypes in both of those B cell developmental stages revealed the presence of IgA2, IgG1, IgG3, and IgG4 at all time points analyzed (Fig. 6*B*); IgE was not found, as expected. We do not believe these observations are due to subset contamination (see Discussion).

To further investigate our observation, we analyzed isotype-specific V gene usage and whether any of the resulting class-switched CDR3s were shared across the B cell subsets. Whereas previously we found an overall V gene usage shift in memory cells relative to the immature and naïve subsets, analysis of V gene usage in the class-switched isotypes revealed that the immature, naïve, and memory cell populations did not have statistically different V gene usage (supplemental Fig. S5).

We then examined if any class-switched CDR3 sequences were shared between the immature, naïve, memory, and plasmacyte cell populations. Because IgM and IgD are co-expressed on naïve B cells, we chose to limit our analysis to CDR3s utilizing IgA, IgG, or IgE (Fig. 6*C*). The percentage of shared CDR3 amino acid sequences for the immature subset was 2.1%, 3.5%, and 7.0% with the naïve, memory, and plasmacyte populations while the naïve subset had overlap percentages of 0.2%, 4.0%, and 2.3% with the immature, memory, and plasmacyte populations, respectively. Our earlier observations of minimal unique CDR3 overlap (see Fig. 3*A*) were mirrored here in that few class-switched CDR3s were observed between the B cell subsets, with only three common CDR3s observed in all four populations (Fig. 6*D*).

In summary, our analysis of isotype distributions within the B cell developmental stages led to the surprising observation that immature and naïve B cells in human peripheral blood may consistently have a small proportion of cells that exhibit isotypes other than the expected IgM and IgD.

## DISCUSSION

Our goal was to study both the dynamics and characteristics of the human immune repertoire to thoroughly define a healthy baseline across B cell development and examine how this changes upon antigen exposure. To this end, we collected longitudinal data from a single individual and examined the CDR3s of flow cytometry-sorted B cell subsets.

Longitudinal data is usually collected under specific immunological challenges (*e.g.* immunization (2) or systemic sclerosis (4)) with few focused on examining healthy fluctuations in the immune repertoire and transition to disease (13). In addition, many of the samples are from bulk cell populations, which limits the conclusions drawn to the strongest signals with weak or unknown contextual reference (2). Alternatively, studies of the human immune repertoire that extensively profiled B cell subsets were based on a single large volume blood draw (7, 8). Our study combines the longitudinal monitoring of both healthy time points and periods of natural infection with subset-specific analysis of the different B cell subsets for a more comprehensive approach to examining the human immune repertoire and its changes in these conditions.

To examine how B cell receptors respond to natural infection, we analyzed changes in CDR3 characteristics. As evidenced in prior studies, we observed the heavy chain CDR3 amino acid length is longest with shorter kappa and lambda chain CDR3 lengths (28–32). The heavy chain CDR3 amino acid usage distribution in our data is also like that previously reported (29, 33). Importantly, our study highlights additional novel aspects of CDR3 biology. First, the differential amino acid usage of the kappa and lambda light chains relative to the heavy chain may indicate different antigen binding characteristics of each light chain in combination with the heavy chain (34), which may enable different specializations in antigen binding (35, 36). Second, both CDR3 length and amino acid usage are predominantly chain-specific as opposed to subset-specific. These observations remain consistent through periods of health and natural infection, suggesting that internal selection pressures (*e.g.* binding affinity for self-antigens) may play an important role in shaping the intrinsic properties of the immune repertoire compared with external sources (*e.g.* foreign antigens).

In examining unique CDR3 amino acid sequences, we found the overlap within each of the three chain types were quite low, even across the different cell types (see Fig. 3). It is important to emphasize that monitoring unique CDR3 RNA sequences longitudinally is a different metric than monitoring B cell clonality at the amino acid level. Because memory cells and plasmacytes are both antigen-experienced populations with high levels of amino acid sequence clonality (37, 38), it follows that the extrapolated lower bound for heavy chain clonotype diversity (calculated as D50 by the iRmap program; see Experimental Procedures) should be lowest for those two cell types. Our data reflects this expectation, with the immature and naïve B cells having the highest diversity and the memory and plasmacyte subsets having the lowest (supplemental Table S8). Hence, finding a low number of common CDR3 sequences in each chain is expected.

Closer examination of the two light chains showed lower unique CDR3 sequence diversity than the heavy chain. This finding is reasonable based on the total numbers of expressed genes involved in recombination. The kappa chains in this individual utilized 40 V genes, five J genes, and one C gene; the lambda chains used 33 V genes, four J genes, and four C genes compared with the heavy chain usage of 48 V genes, six D gene families (data not shown), and six J genes. Therefore, we anticipated lower levels of combinatorial diversity in the two light chains compared with the heavy chain. Also, we found a higher overall level of kappa chain usage than lambda chain as expected (32).

For heavy chain CDR3 sequences, we observed that the number of unique CDR3s in each subset could serve as a proxy for relative B cell subset proportions in human peripheral blood. Additionally, over the course of almost one year, the total number of unique CDR3 predicted amino acid sequences observed across all subsets through all time points was 1,410,924. This total is far lower than other estimates (39), which could be explained by one or more of the following: 1) the lack of universally agreed-upon primer set designs, sequencing platforms, sequencing depth, and analysis pipelines; 2) high efficiency and low permissiveness of healthy immune homeostasis (40); 3) increasing age leading to lower immune repertoire diversity (41–43); and/or 4) preferential usage of V and J genes (7, 23, 44), leading to a lower maximum amount of diversity.

The possibility that the B cell immune repertoire is biased in V gene usage is well known (45, 46), and some genes are consistently selected. In some cases, these preferences are determined prior to foreign antigen exposure (*e.g.* V4–34 and V4–59) while other selected V genes appear to arise in response to some combination of self- and foreign antigens (*e.g.* V3–23 (47)). Therefore, we examined the relationship between VJ gene usage and CDR3 sequence diversity to determine if any additional V genes are preferentially used in a subset-specific manner.

We first observed that, irrespective of health status, a healthy individual's immune repertoire utilizes VJ gene combinations to diversify the resulting CDR3 sequences on the order of one VJ combination to hundreds of different CDR3s. This result aligns with both our observation and others that the relationship between VJ combinations and CDR3 sequences is not directly proportional (48). Note we excluded plasmacytes from this portion of our analysis because their low abundance in peripheral blood (7, 49) may cause uneven sampling of the true diversity of this B cell subset. Interestingly, we found that a single CDR3 sequence may arise from several different VJ combinations, which has not been previously reported.

To further investigate this observation, we examined published datasets (2, 23–25) that also analyzed the human B cell immune repertoire. Irrespective of starting material, primer design, sequencing platform, and data analysis pipeline (Table S9), each of the four studies supports our observation that a single CDR3 can be made from more than one VJ gene combination (supplemental Fig. S6). While the possibility of alignment artifacts and/or PCR chimeras of recombinant immunoglobulin loci cannot be ruled out, we note that these events are found across multiple samples in our study as well as several publicly available independent datasets; thus, it is possible that such recombinations occur *in vivo* through multiple independent events, or through an *in vivo* gene-conversion/rearrangement process similar to that observed in avian systems (50). Additional data on the VJ composition of CDR3 sequences from future studies is crucial to discerning between these possibilities.

Secondly, we observed tight control of V gene usage in each subset for the duration of our study. These subset-specific V gene preferences were maintained irrespective of health status. As a result, we identified several additional heavy chain V genes that warrant further investigation. While some of these genes are involved in human disease (51, 52), other genes would benefit from a more in-depth analysis (V2–5, V3–74, and V6–1). Additionally, we showed several kappa and lambda V genes as differentially expressed between subsets; some have human disease associations validated by the literature (53–56) whereas others merit additional study (KV2D-29, LV10–54, LV2–8, LV3–1, LV3–25, LV4–60, LV4–69).

Another surprising finding of our study is direct evidence of class switching in the immature and naïve B cell subset. This result has not been reported previously and suggests that class switching may occur earlier in human B cell development than previously realized. One of the earliest observations of class switching during early human B cell development was reported by Vogler *et al.* (57) in pre-B cells isolated from children diagnosed with acute lymphoblastic leukemia. Using immunofluorescence assays, they found cytoplasmic IgG and IgA in 0.1–3.6% and 0.7% of leukemic lymphoblasts, respectively, across several patients. Our observations suggest some amount of class switching at the transcriptional level may also occur early in healthy individuals. In addition, the RNA-editing enzyme activation-induced cytidine deaminase (AID) required for both class switch recombination (CSR) and somatic hypermutation (58) is present in both immature and naïve B cells of mice and humans (59–64) with reports that class switching in early B cell development does occur in mice (61, 65, 66).

Another possible explanation for our observation is subset contamination, which we assessed by examining 1) our flow sorting experimental design; 2) the observed class-switched CDR3 sequences; and 3) publicly available datasets from single-cell B cell receptor experiments. For the flow cytometry experiments, we designed our stain panel to maximize both fluorochrome and laser separation for each surface marker. Also, we chose a conservative gating strategy to reduce the inclusion of other cell types and consistently employed backgating to ensure the desired subsets fell squarely within expected clusters. We phenotypically defined immature B cells via surface markers as CD45^+^CD19^+^CD10^+^CD27^−^, whereas the naïve subset was similarly defined except for being CD10^−^. It should be noted that we cannot rule out the possibility that some number of the isotype-switched naïve cells may be the result of isotype-switched immature cells that matured into naïve cells. There is a possibility that transitional B cells were co-sorted with the immature cells as are both CD10^+^CD27^−^ (14, 67). However, considering both the immature and transitional subsets are 1) CD10^+^, which has been accepted as one of the hallmarks of early peripheral blood B cells (67), 2) have the potential to initiate CSR (61–63), and 3) can be ruled out as being recirculating memory B cells (68) by surface CD10 expression, we are confident that we are observing transcriptional evidence of isotype-switching in early human B cells.

Next, we focused on examining the CDR3 sequences of the immature and naive B cells that we identified as class-switched. We expected that the higher the percentage of CDR3 amino acid sequence overlap, the higher the likelihood of contamination from another cell subset. To this end, we first assessed the number of class-switched CDR3s found in the immature and naïve subsets that were present in more than one cell type. Our results indicate that most class-switched CDR3s from the immature and naïve populations were subset-unique.

Finally, we examined single-cell B cell sequencing experiments to determine if existing datasets could either confirm or refute our observation. Analyzing data obtained from individual B cells would eliminate concerns of subset contamination and ensure confident subset assignment of any class-switched B cells based on each cell's specific surface phenotype. However, in most data sets examined, we found that the B cells were not sorted. As a result, it would be impossible to determine with high confidence which B cells were immature, naïve, or memory cells. As a result, any class-switched B cells could not be assigned with certainty to any of the subsets. In those experiments that did sort different B cell populations, isotype surface markers (*e.g.* IgM and/or IgD) were used to phenotypically define their subsets. This would have selected against any class-switched immature or naive cells that may have existed. In other cases, when we tried to access the data, the links were either broken and/or we were unable to locate the datasets within the repositories that contained them. Other published studies that used single cell sequencing to interrogate B cells have not made their data publicly available.

Despite not finding single-cell sequencing datasets that were appropriate for comparison with our study, based on our flow cytometry panel design and class-switched CDR3 sequence analysis, we are confident that, to our knowledge, this is the first reported observation of isotype switching in human early B cell development. If future studies confirm these findings, our observations would give new insights into the developmental and maturation processes of human B cells.

In conclusion, our findings demonstrate the importance of longitudinal data collection when profiling the human immune repertoire. By analyzing these data, we showed 1) V gene usage preferences are remarkably consistent based on foreign antigen exposure; 2) multiple VJ gene combinations may generate the same CDR3 amino acid sequence; and 3) transcriptional evidence of unconventional isotypes in the immature and naïve B cell subsets indicates class switching may occur earlier in human B cell development, a phenomenon only observed previously in mice. Overall, we expect this work to be an in-depth proof-of-principle study for future investigations in which more individuals of different ages, genders, and ethnicities might be studied in the same manner. Such analyses will advance the field of personalized immunology.

## DATA AVAILABILITY

All data generated from this study has been deposited in NCBI's Gene Expression Omnibus (69) with the GEO Series accession number GSE123158 (https://www.ncbi.nlm.nih.gov/geo/query/acc.cgi?acc=GSE123158).

## Supplementary Material

supplemental Fig. S1

Supplemental Figures and Tables

Supplemental Tables S1, S4, and S8

Supplemental Figures and Tables

Supplemental Tables S1, S4, and S8

## Footnotes

^1^ The abbreviations used are:

CDR3: complementarity-determining region 3
V: variable
D: diversity
J: joining
hs-CRP: high sensitivity C-reactive protein.
