# Supplementary material for: Deep Characterization of the Human Antibody Response to Natural Infection Using Longitudinal Immune Repertoire Sequencing
Source: Mol Cell Proteomics. 2019 Nov 25;19(2):278–93. doi: 10.1074/mcp.RA119.001633 (PMC7000125; doi:10.1074/mcp.RA119.001633)
Supplement: supplemental Fig. S1 [file RA119.001633_index.html]

Supplement to Deep Characterization of the Human Antibody Response to Natural Infection Using Longitudinal Immune Repertoire Sequencing | Molecular & Cellular Proteomics

## Supplemental Data

- Supplemental Figures and Tables - Six of nine tables and six figures with corresponding legends as referred to in the manuscript.
- Supplemental Tables S1, S4, and S8 - Three of nine tables corresponding to sorting counts, amino acid usage, and D50 as referred to in the manuscript.
