# Supplementary material for: Deep Characterization of the Human Antibody Response to Natural Infection Using Longitudinal Immune Repertoire Sequencing
Source: Mol Cell Proteomics. 2019 Nov 25;19(2):278–93. doi: 10.1074/mcp.RA119.001633 (PMC7000125; doi:10.1074/mcp.RA119.001633)
Supplement: Supplemental Figures and Tables [file 153617_1_supp_421553_q56f06.pdf]

Deep Characterization of the Human Antibody Response to Natural Infection Using Longitudinal Immune Repertoire Sequencing

Authors: Erin M. Mitsunaga & Michael P. Snyder

List of materials:

1.) Tables

-Table S1. Counts of sorted B cell subsets

-Submitted as an Excel file (Tables_S1_S4_S8.xlsx; ‘Sorting Counts’ tab)

-Table S2. Chain comparisons of CDR3 length

-Table S3. Chain comparisons of overall amino acid usage distributions

-Table S4. Chain comparisons of individual amino acid usage

-Submitted as an Excel file (Tables_S1_S4_S8.xlsx; ‘Amino Acids Usage’ tab)

-Table S5. B cell subset-specific CDR3 counts

-Table S6. Median comparisons of observed unique CDR3s to observed VJ combinations

-Table S7. Subset comparisons of J gene usage

-Table S8. Extrapolated lower bound of CDR3 diversity by D50 calculation

-Submitted as an Excel file (Tables_ S1_S4_S8.xlsx; ‘D50’ tab)

-Table S9. General parameter comparison of B cell sequencing studies

2.) Figures

-Figure S1. Correlation of clinical blood work values with self-reported health state

-Figure S2. One CDR3 can be associated with multiple VJ gene combinations

-Figure S3. Multiple VJ gene combinations are associated with the same CDR3 amino acid sequence

-Figure S4. Heavy chain J gene usage is biased toward J4 through time and health status

-Figure S5. Isotype-specific V gene usage is similar across B cell subsets

-Figure S6. Additional observations of multiple VJ combinations giving rise to one CDR3

Table S1. Counts of sorted B cell subsets

The first row shows the total number of B cells sorted at each time point. The following rows show the quantities of each sorted B cell subset and their percentage of total B cells.

The asterisk on sample H3 indicates that two sorts were performed. The first sort isolated the B cells from all other white blood cells and the second sort separated the bulk B cell sample into the four subsets.

Chain Type Comparison	Corrected *p*-value
Heavy vs. Kappa	**3.0×10^{-4}**
Heavy vs. Lambda	**3.2×10^{-5}**
Kappa vs. Lambda	1

Table S2. Chain comparisons of CDR3 length

Heavy chain CDR3 lengths are significantly different from both kappa and lambda light chain CDR3 lengths as determined by the Kolmogorov-Smirnov test. Bold values indicate $p \leq 0.05$ after multiple hypothesis correction.

	**Immature**	**Naïve**	**Memory**	**Plasmacyte**
**Heavy vs. Kappa**	1	**4.3×10^{-4}**	**0.002**	**0.002**
**Heavy vs. Lambda**	0.10	0.12	0.48	0.61
**Kappa vs. Lambda**	1	**0.001**	**0.002**	**0.003**

Table S3. Chain comparisons of overall amino acid usage distributions

The χ^2 test was used to compare the amino acid usage distributions between the three different chains (row names) based on subset identity (column names). Bold values indicate $p \leq 0.05$ after multiple hypothesis correction.

Table S4: Chain comparisons of individual amino acid usage

Subset-specific individual amino acid usage between the heavy and light chains was compared using the χ^2 test. Bold values indicate $p \leq 0.05$ after multiple hypothesis correction

	Immature	Naïve	Memory	Plasmacyte
**Median % of Total B Cells**	9.3	81.4	7.2	2.1
**Heavy Chain CDR3s**				
Total Overall	752,573	2,185,169	492,905	472,813
Total Unique	209,332	1,112,500	53,583	35,509
Unique to Subset	203,917	1,100,501	43,657	30,385
**Kappa Chain CDR3s**				
Total Overall	1,047,010	1,273,536	819,889	638,326
Total Unique	16,761	31,705	23,608	15,215
Unique to Subset	6,056	17,675	13,431	7,264
**Lambda Chain CDR3s**				
Total Overall	86,702	101,702	58,835	42,927
Total Unique	4,534	6,342	6,743	3,784
Unique to Subset	1,986	3,282	4,586	2,332

Table S5. B cell subset-specific CDR3 counts

The first row shows the median percentage of each B cell subset within the total bulk B cell population. The rest of the table indicates the CDR3 sequence counts over all 24 time points.

	Immature	Naïve	Memory
**Heavy Chain**			
Median # of unique CDR3s per time point	8,094	48,092	4,411
Daily median # of VJ combinations	225	260	214
**Kappa Chain**			
Daily median # of unique CDR3s	2,141	4,114	2,761
Daily median # of VJ combinations	147	146	124
**Lambda Chain**			
Daily median # of unique CDR3s	530	524	706
Daily median # of VJ combinations	52	53	49

Table S6. Median comparisons of observed unique CDR3s to observed VJ combinations

This table shows the comparison of the median number of unique CDR3s to the median number of VJ combinations both by B cell subset and by chain type.

	J1	J2	J3	J4	J5	J6
**Immature vs. Naive**	1	1	1	0.55	1	**0.007**
**Immature vs. Memory**	1	1	1	**0.005**	1	**7.8×10^{-11}**
**Naïve vs. Memory**	1	1	1	1	1	**0.01**

Table S7. Subset comparisons of J gene usage

The χ^2 test was used to compare the J gene usage between the subsets. Bold values indicate $p \leq 0.05$ after multiple hypothesis correction.

Table S8. Extrapolated lower bound of CDR3 diversity by D50 calculation

Each sample's clonality was quantified using the D50 metric developed by iRepertoire (17). The higher the sample's D50 value (maximum value of 50), the higher the sample diversity of B cell clones. The columns are time points and the rows are chain types grouped by subset.

	Starting Material	Primer Design	Sequencing Platform	Data Analysis
**Laserson et al. (2014) Study (2)**	RNA	Self-Designed	Roche 454 GS FLX	Self-Analysis
**DeWitt et al. (2016) Study (42)**	gDNA	Adaptive Biotechnologies	Illumina MiSeq	Adaptive Biotechnologies
**Lee et al. (2016) Study (67)**	RNA	iRepertoire	Roche 454 GS Junior	iRepertoire + Self-Analysis
**Briney et al. (2019) Study (68)**	RNA	Self-Designed	Illumina HiSeq 2500	Self-Analysis
**This Study**	RNA	iRepertoire	Illumina MiSeq	iRepertoire + Self-Analysis

Table S9. General parameter comparison of B cell sequencing studies

This table outlines the different parameters used in each of the reanalyzed B cell sequencing studies compared to this study.

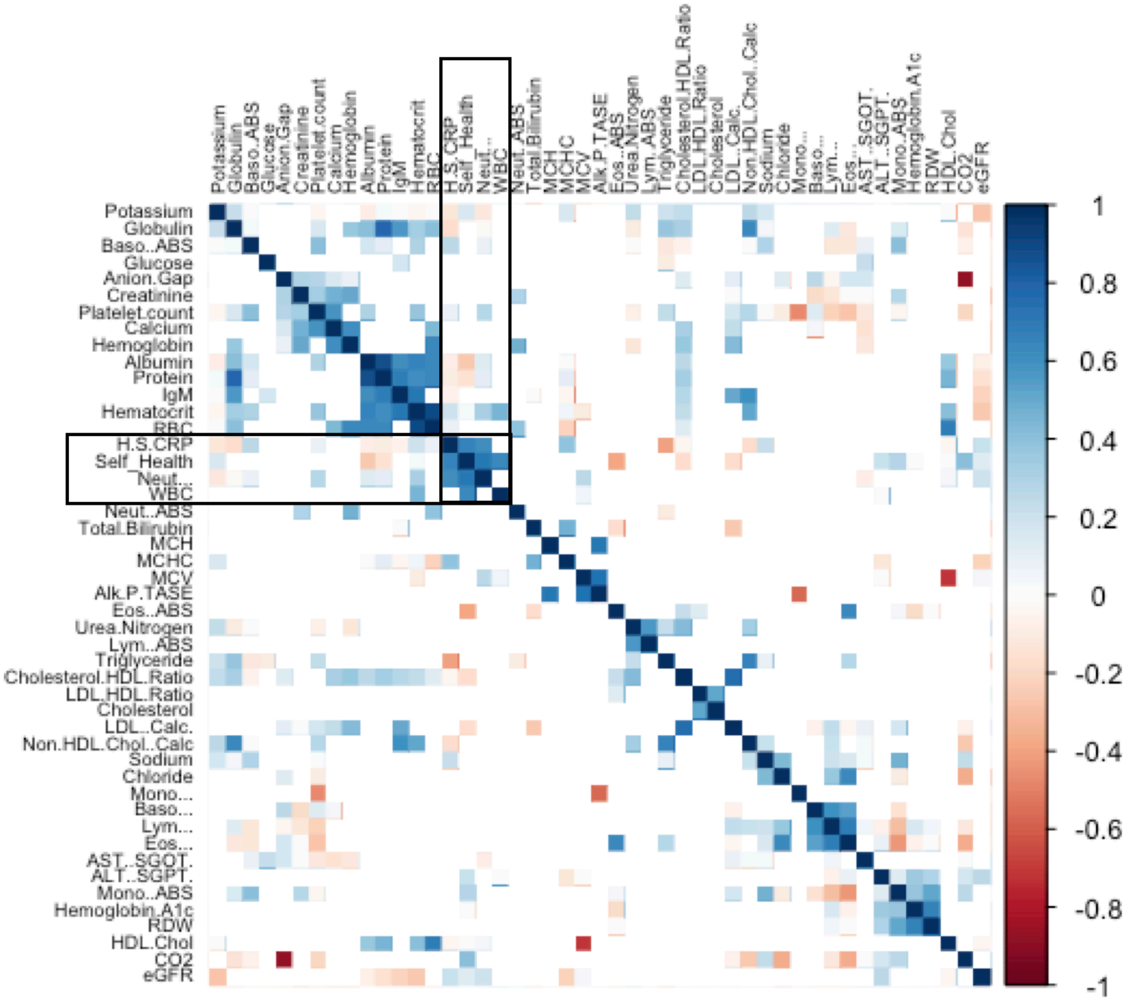

Figure S1. Correlation of clinical blood work values with self-reported health state

High sensitivity CRP, white blood cell count, and neutrophil percentage were most closely correlated with an unhealthy state with a corrected Spearman's rank correlation *p*-value ≤ 0.05. All tests are shown on the x- and y-axes of the correlation plot.

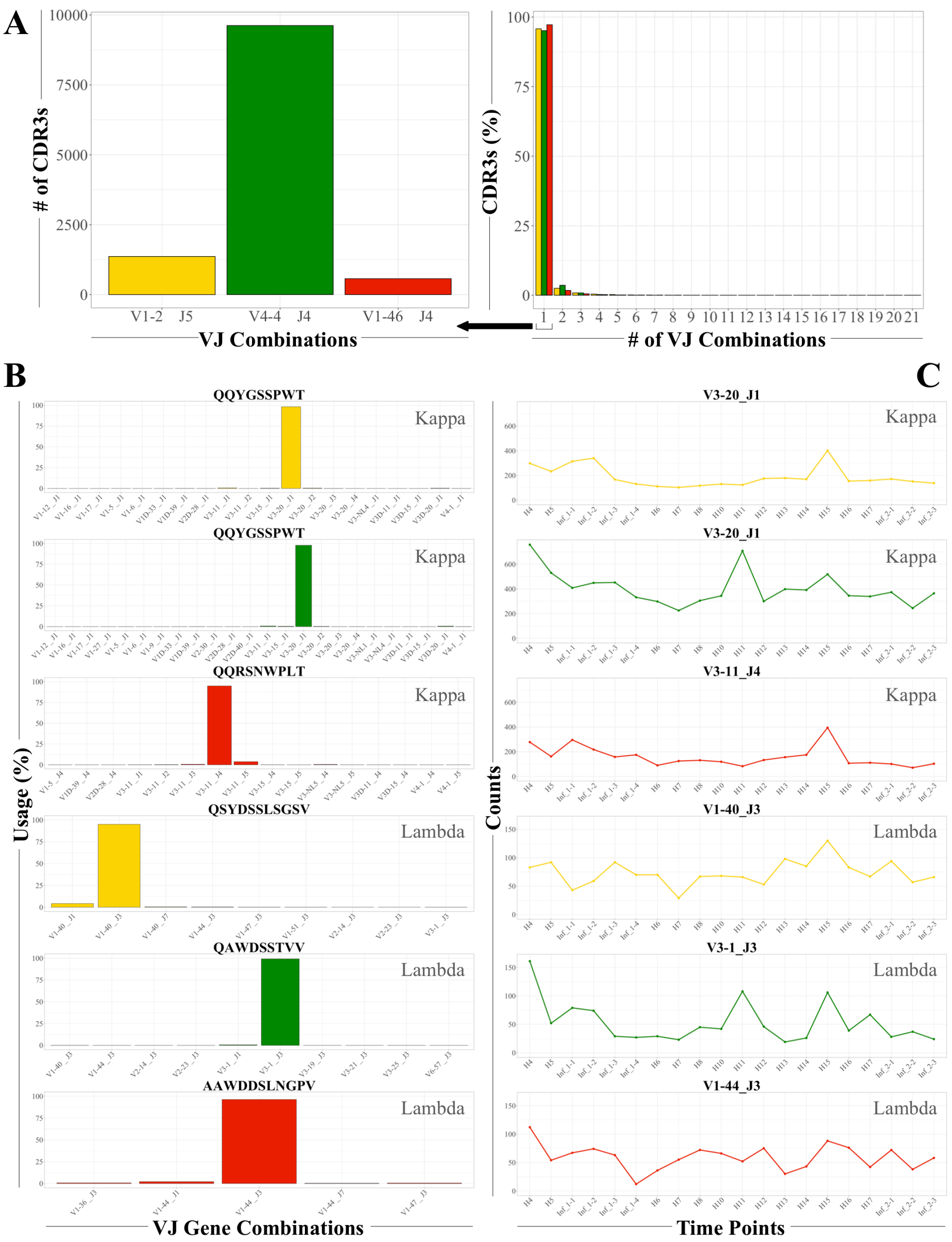

Figure S2. One CDR3 can be associated with multiple VJ gene combinations

The most highly expressed CDR3 across all time points that had multiple VJ gene combinations contributing to overall expression are shown from each B cell subset and light chain type.

(A) The left panel shows the single VJ gene combinations used most by the unique CDR3 sequences from the immature (yellow), naive (green), and memory (red) B cell subsets. The right panel shows the distribution of VJ gene combinations used on the x-axis with the percentage of CDR3 amino acid sequences observed on the y-axis.

(B) The most frequently observed CDR3 amino acid sequences for each subset are displayed in yellow (immature), green (naïve) and red (memory) with the percent observed of each VJ gene combination on the y-axis and the VJ gene combination on the x-axis. The top three bar graphs show the kappa light chain from each subset and the bottom three bar graphs show the lambda light chain from each subset.

(C) The VJ gene combination observed most frequently in panel B was tracked over time to quantify the number of CDR3s that used the same combination. The CDR3 counts observed are on the y-axis and the time points are on the x-axis. The line graphs are shown in yellow (immature subset), green (naïve subset), and red (memory subset). The top three line graphs show the kappa light chain from each subset and the bottom three line graphs show the lambda light chain from each subset.

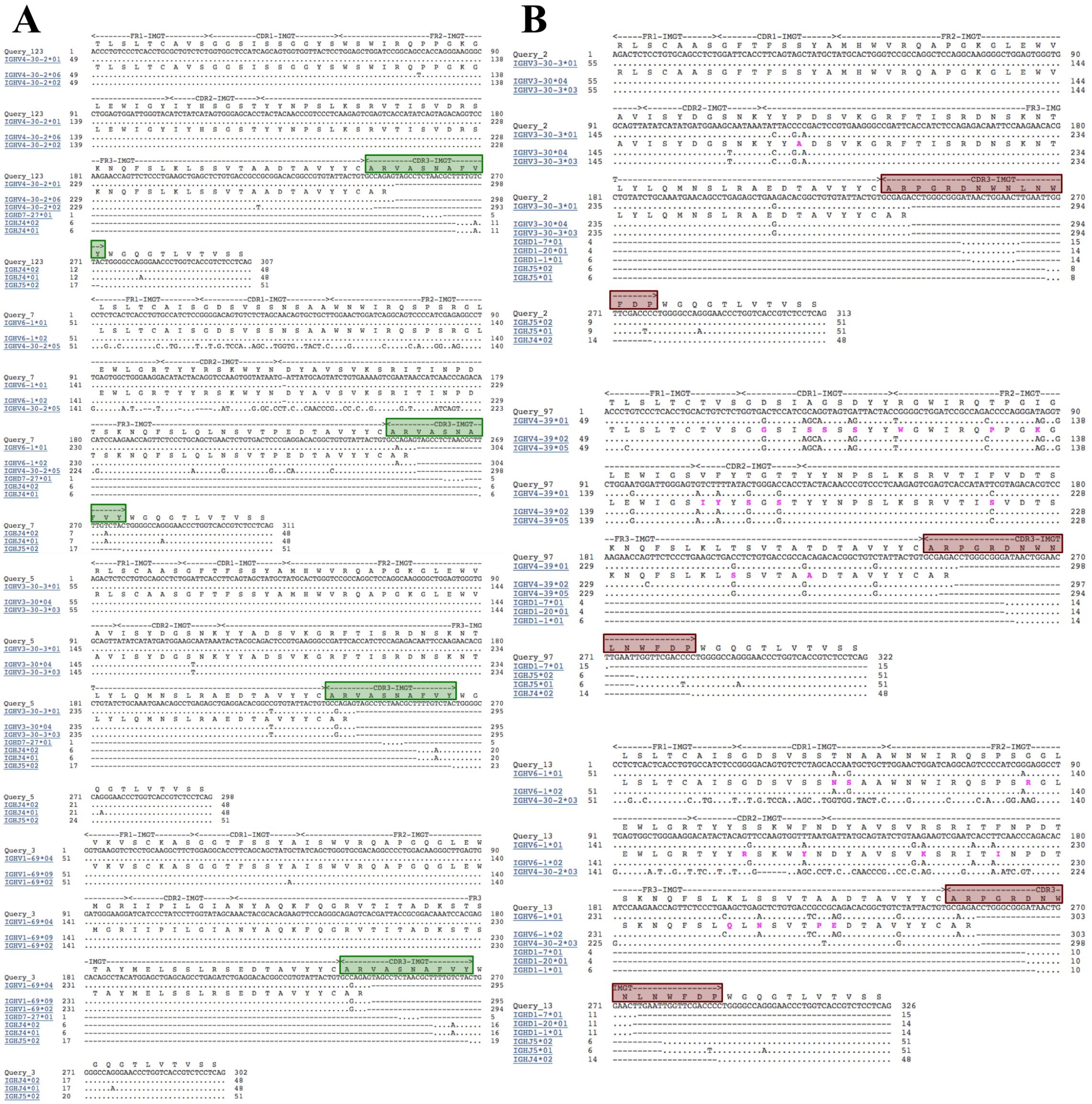

Figure S3. Multiple VJ gene combinations are associated with the same CDR3 amino acid sequence

(A) IgBLAST results for four different VJ combinations that resulted in the same naïve B cell CDR3 amino acid sequence ARVASNAFVY (highlighted in green).

(B) IgBLAST results for three different VJ combinations that resulted in the same memory B cell CDR3 amino acid sequence ARPGRDNWNLNWFDP (highlighted in red).

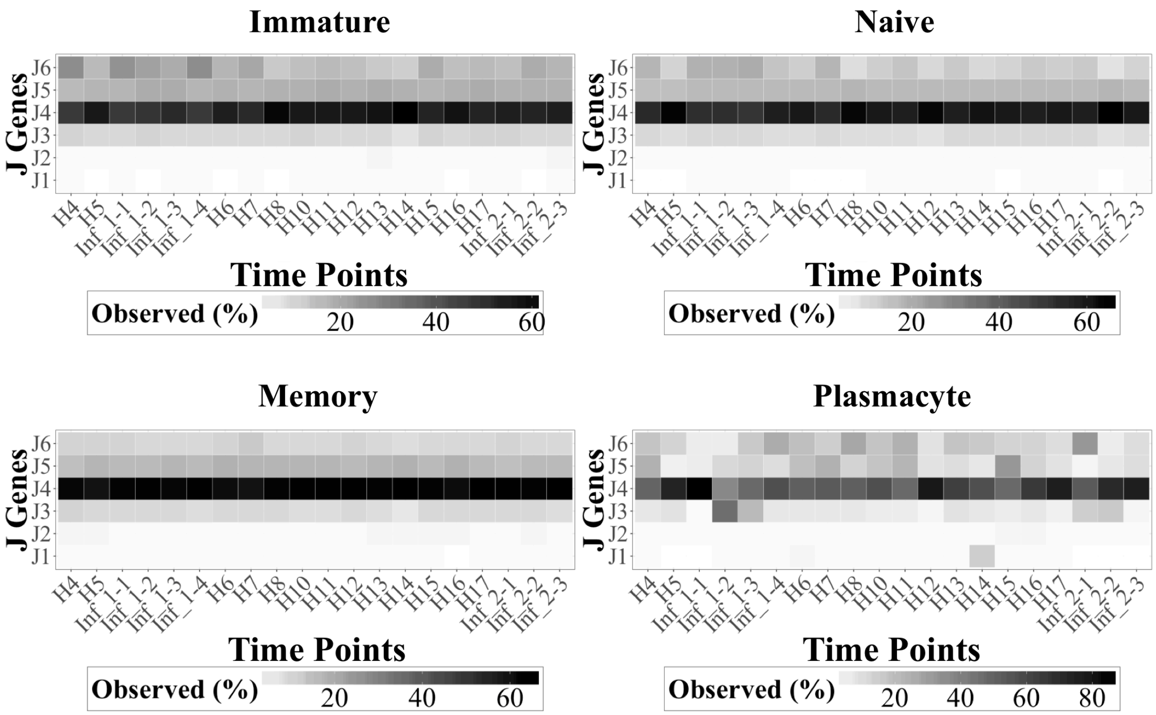

Figure S4. Heavy chain J gene usage is biased toward J4 through time and health status

J gene usage distribution of all six expressed J genes in each of the subsets examined.

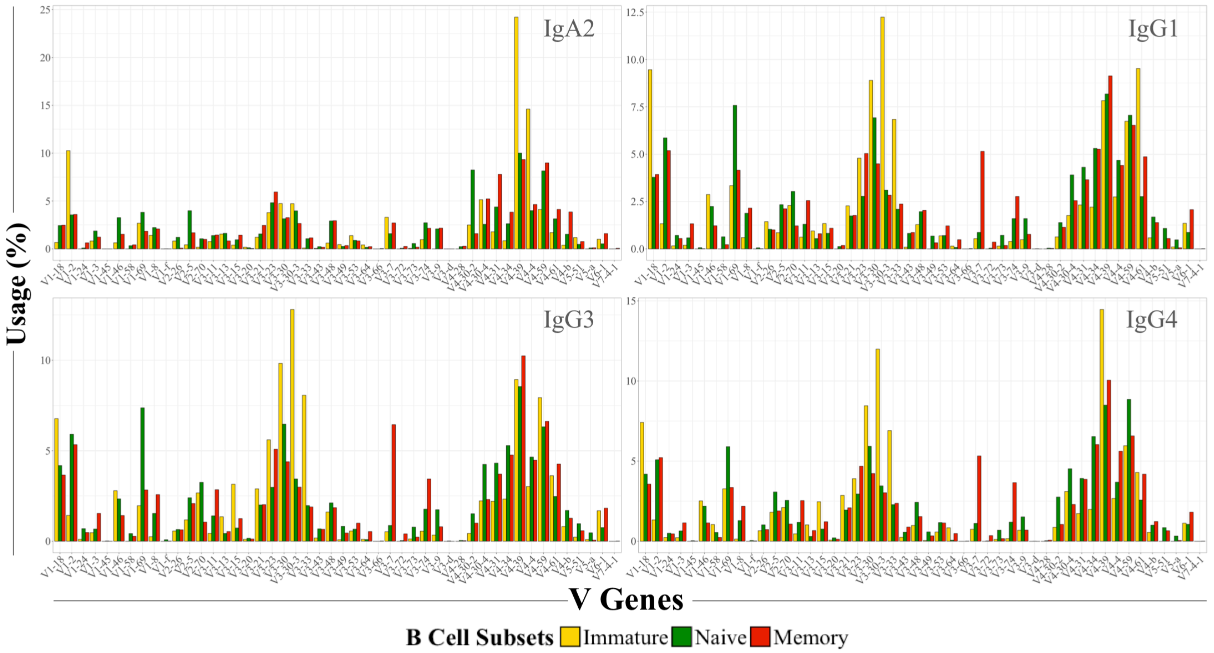

Figure S5. Isotype-specific V gene usage is similar across B cell subsets

A comparison of isotype-specific V gene usage in the immature, naïve, and memory B cell subsets shown in yellow, green, and red, respectively. The y-axis shows percent usage of each V gene and the x-axis shows the individual V genes.

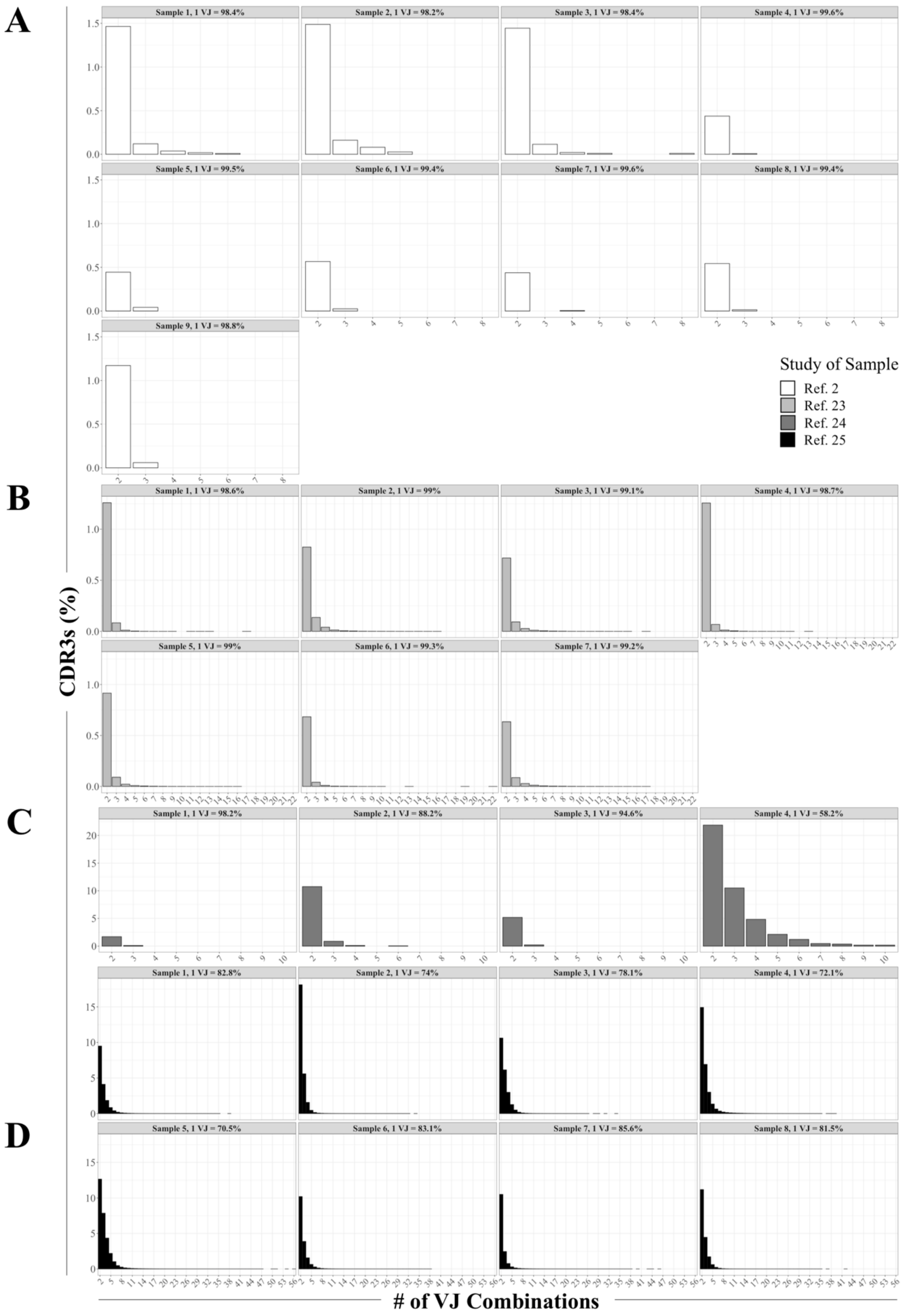
A
Sample 1, 1 VJ = 98.4%
Sample 2, 1 VJ = 98.2%
Sample 3, 1 VJ = 98.4%
Sample 4, 1 VJ = 99.6%
Sample 5, 1 VJ = 99.5%
Sample 6, 1 VJ = 99.4%
Sample 7, 1 VJ = 99.6%
Sample 8, 1 VJ = 99.4%
Sample 9, 1 VJ = 98.8%
Study of Sample
Ref. 2
Ref. 23
Ref. 24
Ref. 25
B
Sample 1, 1 VJ = 98.6%
Sample 2, 1 VJ = 99%
Sample 3, 1 VJ = 99.1%
Sample 4, 1 VJ = 98.7%
Sample 5, 1 VJ = 99%
Sample 6, 1 VJ = 99.3%
Sample 7, 1 VJ = 99.2%
CDR3s (%)
C
Sample 1, 1 VJ = 98.2%
Sample 2, 1 VJ = 88.2%
Sample 3, 1 VJ = 94.6%
Sample 4, 1 VJ = 58.2%
D
Sample 1, 1 VJ = 82.8%
Sample 2, 1 VJ = 74%
Sample 3, 1 VJ = 78.1%
Sample 4, 1 VJ = 72.1%
Sample 5, 1 VJ = 70.5%
Sample 6, 1 VJ = 83.1%
Sample 7, 1 VJ = 85.6%
Sample 8, 1 VJ = 81.5%
# of VJ Combinations

Figure S6. Additional observations of multiple VJ combinations giving rise to one CDR3

(A, B, C, and D) These bar graphs show a closer view of the percentage of CDR3 sequences (y-axis) that are derived from more than one VJ gene combination (x-axis) across four independent studies. The percentage of CDR3s that used only one VJ combination is shown above each bar graph. Study (A) is from reference 2, (B) is from reference 23, (C) is from reference 24, and (D) is from reference 25.
